# Immune Checkpoint Inhibitors in Renal Cell Carcinoma: Molecular Basis and Rationale for Their Use in Clinical Practice

**DOI:** 10.3390/biomedicines11041071

**Published:** 2023-04-02

**Authors:** Francesco Lasorsa, Nicola Antonio di Meo, Monica Rutigliano, Martina Milella, Matteo Ferro, Savio Domenico Pandolfo, Felice Crocetto, Octavian Sabin Tataru, Riccardo Autorino, Michele Battaglia, Pasquale Ditonno, Giuseppe Lucarelli

**Affiliations:** 1 Urology, Andrology and Kidney Transplantation Unit, Department of Precision and Regenerative Medicine and Ionian Area, University of Bari “Aldo Moro”, 70124 Bari, Italy; 2 Division of Urology, European Institute of Oncology, IRCCS, 20141 Milan, Italy; 3 Department of Neurosciences and Reproductive Sciences and Odontostomatology, University of Naples “Federico II”, 80131 Naples, Italy; 4 The Institution Organizing University Doctoral Studies (I.O.S.U.D.), George Emil Palade University of Medicine, Pharmacy, Sciences and Technology, 540139 Târgu Mureș, Romania; 5 Department of Urology, Rush University Medical Center, Chicago, IL 60612, USA

**Keywords:** renal cancer, immunotherapy, immune-checkpoint inhibitors, cancer immune escape, therapy

## Abstract

Renal cell carcinoma (RCC) is the seventh most common cancer in men and the ninth most common cancer in women worldwide. There is plenty of evidence about the role of the immune system in surveillance against tumors. Thanks to a better understanding of immunosurveillance mechanisms, immunotherapy has been introduced as a promising cancer treatment in recent years. Renal cell carcinoma (RCC) has long been thought chemoresistant but highly immunogenic. Considering that up to 30% of the patients present metastatic disease at diagnosis, and around 20–30% of patients undergoing surgery will suffer recurrence, we need to identify novel therapeutic targets. The introduction of immune checkpoint inhibitors (ICIs) in the clinical management of RCC has revolutionized the therapeutic approach against this tumor. Several clinical trials have shown that therapy with ICIs in combination or ICIs and the tyrosine kinase inhibitor has a very good response rate. In this review article we summarize the mechanisms of immunity modulation and immune checkpoints in RCC and discuss the potential therapeutic strategies in renal cancer treatment.

## 1. Introduction

Renal cell carcinoma (RCC) is the seventh most common cancer in men and the ninth most common cancer in women worldwide. In the United States, it is estimated that there will be about 81,800 new cases of kidney cancer (including RCC) and about 14,890 deaths from this disease in 2023 [1].

Recent studies have suggested that RCC can be considered a metabolic disease, as changes in metabolism contribute to the development and progression of this cancer [2,3,4,5,6,7,8,9,10]. One of the main metabolic alterations in RCC is the activation of the hypoxia-inducible factor (HIF) pathway. HIF is a transcription factor that is activated in response to low oxygen levels, or hypoxia. In RCC, HIF is constitutively activated, leading to increased expression of genes involved in glycolysis, angiogenesis, and survival pathways. The metabolic alterations observed in RCC, in association with intratumor heterogeneity, have an important role in the chemo-resistant mechanisms described in this tumor [11,12,13,14]. Furthermore, considering that up to 30% of these patients present metastatic disease at diagnosis, and around 20–30% of patients undergoing surgery will suffer recurrence, we need to identify novel therapeutic targets [15,16,17,18,19,20]. In recent years, the introduction of immune checkpoint inhibitors (ICIs) in the clinical management of RCC has revolutionized the therapeutic approach against this tumor. In this review article, we summarize the mechanisms of immunity modulation and immune checkpoints in RCC and discuss the potential therapeutic strategies in renal cancer treatment [21]. 

## 2. Cancer Immune Surveillance and Escape Mechanisms

**Figure 1:**
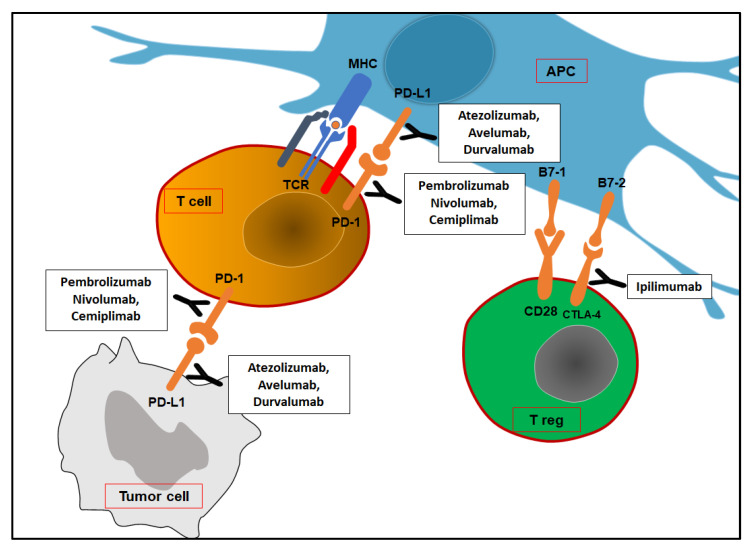
Immune checkpoints and corresponding inhibitors. At tumor sites, PD-1/PD-L1 interaction leads to T cells’ death or inhibition. Treg cells may express CTLA-4 at the lymph nodes, which provides negative feedback signals. APC: antigen-presenting cell; TCR: T cell receptor; MHC: major histocompatibility complex; PD-1: programmed death-1; PD-L1: programmed death ligand-1; CTLA-4: cytotoxic T lymphocyte antigen-4.

Cancer cells develop and proliferate when internal and external checking systems fail [22]. The internal checking system mainly consists of tumor suppressor genes. Apoptosis is eventually activated when uncorrectable genomic errors are detected. The external checking system is mediated by our immune system. There is plenty of evidence about the role of the immune system in the surveillance against tumors. For example, immunodeficient individuals (either primary or secondary) carry a higher risk to develop cancer than immune-competent ones [23]. In addition, some “paraneoplastic syndromes” may develop because of immune responses to cancer (anemia, nephropathy, neuromyopathy, Stauffer syndrome, vasculopathy, coagulopathy, amyloidosis, etc.) [24]. Finally, thanks to a better understanding of immunosurveillance mechanisms, immunotherapy has been introduced as a further treatment of cancer in recent years. Two types of immune responses against cancer cells are known as well as against microbes. Innate immunity represents an early but aspecific reaction, while adaptative immunity provides a very specific but delayed response. In this scenario, the ultimate cause of cancer can be explained as the uncontrolled proliferation of cells that have evaded immune system attack. Phagocytic cells (neutrophils and macrophages), dendritic cells (DCs), NK cells, and other lymphoid cells are the main effectors of innate immunity. A series of elements have been documented to interfere with DC maturation [25,26,27]. Notably, IL-35 has been reported to lower the expression of the costimulatory molecules CD40, CD80, and CD86 as well as HLA-DR and CD83. Similarly, IL-6 has been noted to inhibit DC maturation by reducing MHC class II and CD86 expression. CD47 on tumor cells may block macrophage phagocytosis by binding SIRP-α (signal regulatory protein α—an inhibitory receptor on phagocytes) [28]. Recently, CD47 expression has been associated with more aggressive phenotypes of clear cell renal cell carcinoma (ccRCC) and poorer patient prognosis [29]. NK cells destroy several tumor cell types, particularly those with decreased MHC I expression or that express ligands for NK-activating receptors. NK cells are thought to be the first line of protection against blood-borne metastatic tumor cells. Patients with metastatic disease show aberrant NK cell activity, and low NK cell levels may predict impending metastases. There are two types of adaptive immune responses: cellular (T cells) and humoral (B-cells). Besides blocking the function of their target, antibodies produced by B cells may enhance the elimination of their target in a process named “antibody-dependent cell-mediated cytotoxicity” (ADCC). To be activated, adaptive immune cells demand antigen-presenting cells (APCs) such as dendritic cells and their antigen-presenting structure represented by the major histocompatibility complex (MHC). Antigen peptides in MHC are recognized by T cell receptors; CD8 T cells bind class I MHC, whereas CD4 T cells bind class II MHC. CD4 and CD8 are T cell coreceptors that bind non-polymorphic regions of MHC molecules. Moreover, CD28 co-stimulation is necessary for T cell activation since CD28 binds B7-1 (CD80) and B7-2 (CD86) on activated macrophages, dendritic cells, and B lymphocytes. Ongoing mutations may lead cancer cells to reduce or even to turn off tumor-specific antigens’ expression such as class I MHC, β-2 microglobulin, or components of the antigen-processing machinery (i.e., tapasin and TAP) [30,31]. A low tumor mutation burden (TMB) has already been associated with a reduced response to immunotherapy since tumor cells tend to express fewer neoantigens on MHC to immune cell effectors. In line with this, the loss of the most immunostimulatory neoantigens is thought to be a mechanism of immunoediting that may pave the way to resistance to immunotherapies [32]. Effector T cells’ homing to tumor sites is impaired as well. VEGF promotes the growth of aberrant blood vessels, and it downregulates adhesion molecules’ expression (such as ICAM-1 and VCAM-1), which limit T cells’ extravasation to tumor microenvironment (TME), as does endothelin B receptor overexpression [33,34,35]. The expression of “immune checkpoint” molecules also contributes to the active suppression of immunological responses (Figure 1) [36].

Most human solid cancers express PD-L1, a B7 family protein that binds T cells’ inhibitory receptor PD-1 (programmed death-1). In tumor sites, PD-L1/PD-1 interaction leads activated immune cells to either die or lose their function. IFN-γ produced by activated T cells may block cancer cell proliferation by interfering with DNA duplication. At the same time, IFN- γ induces PD-L1 expression on cancer cells. However, APCs may express PD-L1 to avoid T cell over-activation. Tumor-infiltrating dendritic cells are known to express PD-L2 (also called B7-DC), which is another ligand of PD-1 (not expressed by most human cancer cells) [37,38]. At the lymph node level, activated T cells may express CTLA-4 (cytotoxic T lymphocyte antigen-4), which provides negative feedback signals for T cell activation. The presentation of tumor antigens by APCs in the absence of robust innate immunity and consequently with low levels of B7 costimulators has been proposed as a potential explanation for the participation of CTLA-4 in this process. Autoimmune responses have been described as a side effect of immune checkpoint blockade therapy. Other immune checkpoint molecules have been discovered (B7-H3, B7-H4, VISTA, PD-1H, Tim-3, LAG3, TIGIT, etc.), and clinical trials have already tested their clinical relevance [39,40]. Moreover, tumor cells and tumor-associated macrophages (TAMs- M2 phenotype) may release products (TGF-β, IL-10, VEGF, prostaglandin E2, etc.), which are able to block the proliferation and functions of lymphocytes and macrophages. M2 phenotype depends on alternative macrophage activation by type 2 CD4 T cells (T_H_2)’ cytokines (IL-4 and IL-13). TAMs express arginase and IDO as well as PD-L1 and PD-L2, whose expression is enhanced by macrophage’s chemokine CXCL8 [41,42]. A recent study investigated the role of MUC1 in ccRCC. Overexpression of the anaphylatoxin C3a (C3aR) and C5a (C5aR) receptors was seen in MUC1-expressing ccRCCs (MUC1^H^). MUC1^H^ ccRCC characterized by high microvessel density, high M2-TAM (IDO+) infiltrates, and altered metabolism can be recognized as an immunologically silent subset of renal cancer [43,44,45,46].

Cancer cells may recruit myeloid-derived suppressor cells (MDSCs) to inhibit the immune responses at tumor or lymph node sites. MDSCs are bone marrow cells whose process of differentiation into APCs is disrupted. They may accumulate also at sites of chronic inflammation. Their recruitment depends on proinflammatory mediators such as prostaglandin E2, IL-6, VEGF, and complement fragment C5a. At tumor sites, MDSCs release IL-10 and free radicals such as peroxynitrite, and they also express indolamine 2,3 dioxygenase (IDO1) and arginase-1 (ARG1). IDO1 transforms L-tryptophan (TRP) to kynurerine (KYN), whereas arginase-1 reduces L-arginine availability. ARG1-expressing cells also include M2-TAMs and T regs [47]. Reduced levels of these amino acids impair T cell proliferation [48,49]. In addition, regulatory T cells (Treg) may dampen T cell responses at tumor sites and lymph nodes. To date, different mechanisms of Tregs’ immunosuppressive activity are known besides the expression of inhibitor checkpoint molecules. Tregs may secrete IL-2, IL-10, TGF-β, adenosine, granzyme, and/or perforin, thus limiting the activity of effector CD8 T cells [50,51,52]. This has been supported by the finding that reducing the Treg population significantly slows tumor growth and raises the proportion of CD8 T cells in tumor sites [53].

## 3. Immunometabolic Rewiring of Cancer

Current evidence points out that crosstalk between cancer metabolic reprogramming and anti-tumor immune response occurs [54,55]. Cancer cells can reduce immune responses by competing for and depleting vital nutrients, increasing oxygen consumption, and producing reactive nitrogen and oxygen intermediates. The proliferation, differentiation, activation, and function of immune cells may also be significantly influenced by aberrant metabolites and intermediates in the TME. Interestingly, immune cells may activate different metabolic pathways according to their functional state (T cells above all) [56]. The primary source of energy for activated neutrophils, M1 macrophages, and iNOS-expressed DCs is glycolysis. Oxidative phosphorylation (OXPHOS) from fatty acid oxidation is the main energy source of M2 macrophages and Tregs [57]. Competitive uptake of glucose by cancer cells may inhibit the function of tumor-infiltrating T cells. An inverse relationship between GLUT1 expression and infiltrating CD8 T cell number has been outlined in RCC specimens [58]. Aberrant aerobic glycolysis of tumor cells (Warburg effect) results in lactate accumulation in TME and its acidification. An acidic TME has been shown to limit the activity of both T cells and myeloid immune cells. Lactate may reduce IFN-γ production by NK cells by silencing the nuclear factor of activated T cells (NFAT) signals [59]. Glutamine plays a crucial role in many activities of immune cells, including cell proliferation, antigen presentation, phagocytosis, production of cytokines, NO, and peroxide. Subsequently, these may be dampened by glutamine deprivation by cancer cells. Increased lipogenesis for membrane phospholipids and signaling molecules is frequently observed in tumor cells. Hence, it has been demonstrated that metabolic reprogramming causes tumor-infiltrating myeloid cells (including MDSCs, DCs, and TAMs) to skew towards immunosuppressive and anti-inflammatory phenotypes. This may be caused by the aberrant accumulation of lipid metabolites (such as short-chain fatty acids, long-chain fatty acids, cholesterol, etc.). Cholesterol concentration in cancer cells has been shown to be higher than in immune cells: a high amount of sterol promotes immune checkpoint molecules’ expression [60]. A high rate of cholesterol esterification in the tumor can impair immune responses; as a result, disrupting cholesterol esterification to increase the concentration of cholesterol in immune cells’ plasma membranes may promote the proliferation of these cells and enhance their ability to function as effectors. Therefore, future deeper insights into immune cells’ metabolism might be useful to develop metabolism-targeting therapies, enhancing the possibility for immunotherapy synergy. 

## 4. Immunotherapy in RCC

**Figure 2:**
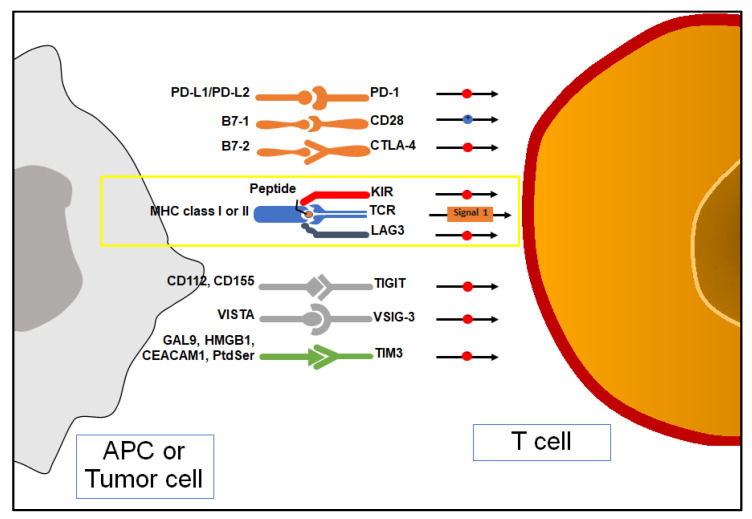
Antigen presentation, co-stimulation, and immune checkpoint inhibition (ICIs) of T cell. APC: antigen-presenting cell; MHC: major histocompatibility complex; PD-1: programmed death-1; PD-L1/PD-L2: programmed death ligand-1/2; TIM-3: T cell Immunoglobulin and mucin domain-containing 3; Gal9: galectin9; HMBGB1: high-motility group box-1; CEACAM1: carcinoembryonic antigen cell adhesion molecule; Ptdser: phosphatidylserine; TIGIT: T cell Immunoreceptor with immunoglobulin and ITIM domain; TCR: T cell receptor; CTLA-4: cytotoxic T lymphocyte antigen-4; LAG-3: lymphocyte activating-gene-3; VISTA: V-domain immunoglobulin suppressor of T cell activation; VSIG-3: V-set and immunoglobulin domain containing protein 3.

Renal cell carcinoma (RCC) has long been thought chemoresistant but highly immunogenic. In the 1960s, spontaneous remission of metastatic patients was observed after the surgical removal of primary tumors [61]. Immunotherapy agents do not directly destroy their targets, but they stimulate immune responses to destroy them. IL-2 and IFNα2b have been the first immunotherapy regimens used to treat metastatic RCC until the development of new agents in 2005 [62,63]. With effects on both effector and regulatory T cells, IL-2 was already known to promote T cell proliferation and differentiation. Therefore, high dose- IL-2 was authorized in 1992 for the treatment of metastatic RCC. Because of the pharmacokinetics of a pegylated form (Bempegaldesleukin), high doses of IL-2 may be avoided [64]. Currently, the administration of IFN is approved in combination with bevacizumab for metastatic RCC [65,66]. Advanced RCC systemic therapy has evolved over the past 20 years from a non-specific immune strategy (the cytokine era) to targeted therapy against vascular endothelial growth factor (VEGF), VEGF receptor (VEGFR), and immune checkpoint inhibitors (ICIs) [67] (Figure 2).

## 5. Mechanism of Action of ICIs

### 5.1. Cytotoxic T-Lymphocyte Antigen 4 (CTLA-4)

Costimulatory signals are necessary for T cell activation. This is achieved when B7-1 and B7-2 on APCs bind CD28 on naïve T cells. CTLA-4 is a member of the CD28 receptor family, so, when binding to B7, it negatively regulates T cell activation. The engagement of CTLA-4 and its ligand B7 activates the serine/threonine phosphatase PP2A, thus reducing TCR- and CD28-associated signaling pathway (reduced AKT activity) [68,69]. A strong association between CTLA-4 and T cell infiltration has been observed in several cancer tissues, where it has been reported to be significantly expressed (including in ccRCC) [70]. 

### 5.2. Programmed Cell Death Protein-1 and Its Ligand (PD-1/PD-L1) 

PD-1 and its ligands PD-L1 and PD-L2 may downregulate the TCR signaling pathway. It has been noted that PD-1 expression increases upon the exposure of naïve T cells to antigens, and it decreases as the antigen disappears. In the case of persistent antigen exposure, PD-1 is continuously highly expressed as it happens in ccRCC. Hence, PD-L1 and PD-L2 are highly expressed in primary and metastatic sites of ccRCC [71]. After their binding, the PI3K-AKT and the RAS-MEK-ERK pathways will be downregulated. This will lead to significantly reduced T cell proliferation, cytotoxic molecule production, and killing capacity. In contrast, regulatory T cells’ maturation and function will be kept [72,73,74].

A recent study highlighted that PD-L1 level was reduced in ccRCC characterized by increased expression of MUC1 [44].

The increased nuclear grade was associated with PD-L1 expression. In metastatic ccRCC, it may also be positively linked to higher sarcomatoid features, advanced T stage, primary tumor size, International Metastatic RCC Database Consortium (IMDC), or Memorial Sloan Kettering Cancer Centre (MSKCC) risk scoring, as well as the occurrence of numerous metastases, although these correlations are less evident [75,76].

### 5.3. T Cell Immunoglobulin and Mucin Domain-Containing 3 (TIM-3)

T cell immunoglobulin and mucin domain-containing 3 (TIM-3) is a type I trans-membrane protein with coinhibitory activity that has been found on IFN-γ producing T cells, FoxP3+ Treg cells, and innate immune cells (macrophages and dendritic cells) [77]. TIM-3 locus maps on chromosome 5q33.2 in the human genome near the IL-4 gene cluster [78]. Previous studies have already demonstrated the association of TIM-3 with autoimmune and allergic disorders in both murine and human models. Effector T cells produce IFN-γ, which promotes both direct anti-tumor activity and expansion of myeloid-derived suppressor cells (MDSCs). MDSCs produce high levels of Galectin-9 (Gal9); by binding to TIM-3, effector CD8+ T cells lead to apoptosis [79]. Moreover, TIM-3+ FoxP3+ Tregs release high amounts of molecules with an inhibitory effect on effector T cells (i.e., IL-10, etc.). Dendritic cells have been shown to express more TIM-3 in a tumor microenvironment (TME) than in healthy tissue. High-mobility group box 1 (HMBG1) allows the tumor-derived nucleic acids transport into dendritic cells. Upon binding to HMBG1, TIM-3 prevents the latter endosomal trafficking, thus limiting innate immune responses to tumor-derived nucleic acids [80]. To date, other TIM-3 ligands have been identified such as carcinoembryonic antigen cell adhesion molecule (Ceacam1) and phosphatidylserine (PtdSer). Therefore, overexpression of TIM-3 is associated with T cell exhaustion (T cell suppression and dysfunction) in tumor-associated leukocytes (TILs). TIM-3 expression has been found to be closely linked to PD-1 expression. In ccRCC, VHL loss results in an increased expression of VEGF, which has recently been associated with the upregulation of PD-1 and TIM-3 on CD8-T cells [81]. Granier et al. has already demonstrated that RCC patients with tumor-infiltrating CD8 cells co-expressing PD-1 and TIM-3 experienced a more aggressive phenotype, as shown by a high Fuhrman grade, a larger tumor size, and more advanced TNM and UISS (UCLA Integrated Staging System) scores [82]. Targeting both the TIM-3 and PD-1 pathways simultaneously is believed to be more efficient than targeting either pathway alone. Nonetheless, some data suggest that TIM-3 may even exert co-stimulatory effects on CTL and other immune effectors [83,84].

### 5.4. T Cell Immunoreceptor with Immunoglobulin and ITIM Domain (TIGIT) 

T cell immunoreceptor with immunoglobulin and ITIM domain (TIGIT) is a novel promising co-inhibitory receptor that is upregulated in NK cells, activated T cells, memory T cells, and FoxP3+ T regs [85,86]. It belongs to the poliovirus receptor (PVR) family. TIGIT competes with the activator receptor CD226 for the same ligands CD155 (PVR) and CD112 (PVRL2) that are expressed by tumor cells and APCs in the TME. Different mechanisms of action have been described. First, because of the engagement of TIGIT by CD155, inhibitory signals in T and NK cells are triggered. TIGIT binds to CD155 on APCs to increase the production of IL-10 and reduce the production of IL-12, which indirectly suppresses T cells (tolerogenic dendritic cells). At the same time, T regs’ immunosuppressive functions are enhanced. Ligation of TIGIT promotes the release of inhibitory molecules IL-10 and Fgl2 (fibrinogen-like protein 2) by T regs. In TIGIT+ T-regs, TIGIT upregulates TIM-3 expression, so it synergizes with TIM-3 and LAG-3 [87,88]. TIGIT can decrease CD8 T cell proliferation and immunosuppression by interacting with the PD-1/PD-L1 pathway as well [89,90]. In this regard, Hong et al. investigated the biological functions of TIGIT and PD-1 in the development, invasion, and metastasis of RCC, as well as their relationship with the clinicopathological features of RCC [91]. 

### 5.5. Lymphocyte Activation Gene-3 (LAG-3) 

Lymphocyte activation gene-3 (LAG-3) is expressed on Treg cells, NK cells, CD4 and CD8 T cells as a response to persistent antigen stimulation [92]. It shows ammino acid homology to CD4. LAG-3 binds with higher affinity to the peptide-MHCII complex than CD4, thus limiting CD4 T cells’ activation [93,94]. CD8 T cells are inhibited because of LAG-3 recruitment, although mechanisms have not been elucidated yet; other ligands may likely exist (such as Galectin-3, lectin LSECtin, and fibrinogen-related protein FGL-1) [95,96,97]. LAG-3+ Treg cells release inhibitory cytokines (such as IL-10 and TGF-β), which further suppress antitumor T cell’s activities [98]. The most common inhibitory receptor combination in CD4 and CD8 T lymphocytes in ccRCC tissues was identified to be LAG-3 and PD-1. Co-expression of LAG-3 and PD-1 is associated with intratumoral T cell dysfunction [99]. LAG-3 was upregulated in response to PD-1 inhibition, and enhanced IFN release was observed after dual blockade of both during in vitro stimulation. Zelba et al. indicated that PD-1 and LAG-3 co-blockade might be a potential treatment option for advanced ccRCC since these inhibitor receptors (IRs) were found to be similarly expressed. These results were obtained from samples of primary RCC tumors, and IR expression may also vary according to the metastatic sites [100,101]. Metalloproteinases may remove LAG-3 and TIM-3 from cell surfaces, and their soluble forms are then released [102,103].

### 5.6. Indoleamine 2,3-Dioxyegenase 1 (IDO1)

Indoleamine 2,3-dioxyegenase 1 (IDO1) catalyzes the rate-limiting step in the KYN pathway. Besides MDSCs, IDO1 has been found expressed in mature DC, as well as in macrophages, cancer, endothelial and stromal cells in ccRCC [48,104]. TRP deprivation has been noted to induce apoptosis in T cells to limit their proliferation. TRP catabolism causes autophagy in T cells and inhibits the immunomodulatory kinases mTOR and protein kinases C [105]. Additionally, further studies have revealed that KYN inhibits antitumor immune responses and stimulates T cell differentiation into FoxP3+ T reg cells via aryl hydrocarbon receptor (AHR) [106]. For these reasons, IDO1 is consequently thought to be a possible cancer immunological checkpoint.

### 5.7. V-Domain Immunoglobulin Suppressor of T Cell Activation (VISTA)

V-domain immunoglobulin suppressor of T cell activation (VISTA or B7-H5) is overexpressed in different tumor cells and in immune cells in TME. By interacting with inhibitory receptors on T cells, VISTA limits their proliferation and activation, whereas it induces FoxP3 expression. Hence, VISTA expression is associated with a state of tumor immunosuppression [107,108]. On the other hand, VISTA may play a stimulatory checkpoint role in anti-cancer immunity in specific malignancies (i.e., esophageal, gastric, liver, and ovarian cancers). VISTA was discovered to be markedly elevated in ccRCC, even beyond the level of PD-L1. CD14+ HLA-DR+ macrophages were revealed to express higher levels of VISTA in ccRCC [109]. A more significant efficacy was noted when anti-VISTA therapy was combined with PD-1 or CTLA-4 blockade than in monotherapy [110]. At present, ten members of the B7 family have been identified: B7-1 (CD80), B7-H1 (PD-L1), B7-DC (PD-L2), B7-H2, B7-H3, B7-H4, B7-H5, B7-H6, and B7-H7. 

They are considered to form receptor–ligand networks, which may regulate immune responses differentially in a context-dependent manner in different human malignancies [74].

## 6. Use of Immune Checkpoint Inhibitors (ICIs) in Clinical Settings

**Figure 3:**
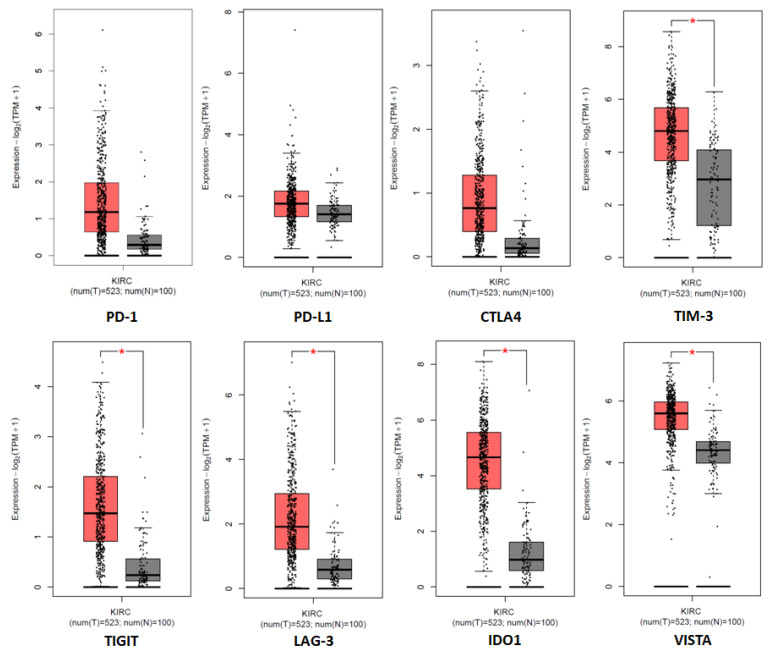
Tissue expression of the immune checkpoint genes in the cancer genome atlas (TCGA) clear cell RCC patient cohort (KIRC). * denotes *p* < 0.05.

Considering the increased expression of different immune checkpoint molecules in ccRCC (Figure 3), in recent years, many clinical trials have been conducted to evaluate the use of ICIs as a novel therapeutic approach in this tumor.

A phase II clinical trial (NCT00057889) was conducted to evaluate the efficacy of ipilimumab (CTLA-4 inhibitor), which became the first immune checkpoint inhibitor (ICI) used to treat patients with metastatic ccRCC [111]. For the first time, tumor remission was confirmed in patients who did not respond to IL2 treatment. A phase III clinical trial (CheckMate 025, NCT001668784) compared everolimus to nivolumab (PD-1 inhibitor) in patients with advanced ccRCC. Patients’ overall survival (OS), overall response rate (ORR), and treatment-related adverse events (AEs) were demonstrated to improve with the PD-1 inhibitor. Therefore, nivolumab significantly outperformed everolimus as a second-line treatment for advanced ccRCC in terms of survival and safety [112,113]. The first combination immune blockade treatment for ccRCC was nivolumab plus ipilimumab, which was first assessed for efficacy and safety in a phase I trial (Checkmate 016, NCT01472081) [114]. Different doses of the combination were used, but the lower dose ipilimumab combination appeared less toxic. In an international multicenter phase 3 trial (CheckMate 214), where patients were randomly assigned, a combination of nivolumab plus ipilimumab was found to have a higher ORR and longer progression-free survival (PFS) in intermediate and poor-risk patients than sunitinib [115,116]. Phase II clinical trial KEYNOTE-427 (NCT02853344) assessed pembrolizumab (another PD-1 inhibitor) as a single therapeutic agent for ccRCC, and it appeared as tolerable as for patients with other tumor types [117]. Phase II trial IMmotion 150 (NCT01984242) evaluated atezolizumab (PD-L1 blocker) as a first-line therapy for RCC [118]. Another PD-L1 blocker that has recently been developed is spartalizumab [119]. 

In recent years, several trials combining ICIs with anti-VEGF therapy have taken place with different results. Given the weak results in terms of OS within phase III trial IMmotion151 (NCT02420821), bevacizumab (anti-VEGF) plus atezolizumab failed to be approved by FDA for advanced RCC [120]. Avelumab (anti-PD-L1) plus axitinib (a tyrosine kinase inhibitor-TKI) were evaluated in the phase III trial JAVELIN Renal 101 (NCT02684006). Compared to the sunitinib group, patients receiving combination therapy had higher median PFS and ORR; however, long-term follow-up data are still needed to demonstrate the true benefit [121]. The results of phase III trial KEYNOTE-426 (NCT02853331) led to the approval by the FDA of pembrolizumab plus axitininb as first-line therapy for advanced ccRCC [122]. In a randomized controlled phase III trial CLEAR (NCT02811861), a longer median PFS and superior OS benefit were achieved in the lenvatinib (TKI) plus pembrolizumab group than in sunitinib one [123]. Nivolumab and cabozantinib (TKI) together also showed greater oncological efficacy than sunitinib [124]. Finally, in a COSMIC-313 phase 3 randomized controlled study, the triple regimen of cabozantinib, nivolumab, and ipilimumab was evaluated as the first-line systemic treatment for metastatic ccRCC [125]. In turn, although both groups receive considerable benefits from ICI combination regimens over sunitinib, PD-L1+ patients seem to respond to anti-PD-1/PD-L1 drugs more favorably than PD-L1- patients [75]. Several clinical trials are ongoing or have evaluated the use of new ICIs for the treatment of renal cell carcinoma (Table 1). 

CD8+ T cell activation and tumor growth inhibition were induced in ccRCC patients by IMP231, a recombinant soluble LAG-3Ig fusion protein [126,127]. On the other hand, despite having a good tolerability profile, sabatolimab (anti-TIM3) did not produce significant benefits in advanced solid cancers, either in monotherapy or in combination with spartalizumab [128,129]. Navoximod (IDO1 inhibitor) plus atezolizumab were evaluated in a phase I trial involving seven patients with advanced RCC (ORR 43%) [130]. There are current clinical trials for VISTA and PD-L1 blockade together in advanced cancers (NCT02812875) [131]. Different results were achieved in patients with non-clear-cell RCC (nccRCC) with ICIs [132,133]. Histology represented one of the main factors affecting the outcomes: better responses were obtained in papillary and sarcomatoid dedifferentiated tumors, whereas poorer responses were found in chromophobe renal cancers. Nevertheless, rarer histologic subtypes (such as translocation renal cell carcinoma) and aggressive renal cancers (such as collecting duct tumors) were documented to respond to ICI-based therapy [134,135,136]. To further understand the true level of efficacy of ICI monotherapy in the nccRCCs, more prospective trials are required, since better outcomes were associated with ICI combination regimens. Another intriguing role of immunotherapy may be its pre-operative administration in metastatic and neoadjuvant settings. Meaningful reductions in the primary tumor were demonstrated by first-line combination regimens in the metastatic setting, thus facilitating cytoreductive nephrectomy. Early evidence from the neoadjuvant setting supports the use of VEGFR-TKIs either alone or in combination with ICIs, whilst the outcomes of single neoadjuvant ICI are disappointing [137,138].

## 7. Conclusions

Despite the clinical success of current anti-CTLA-4, anti-PD-1/PD-L1 agents, a significant number of RCC patients remain unresponsive or even develop resistance. In such a complicated tumor immune environment, blocking a single checkpoint may result in the activation of other immune modulators. Targeting novel ICIs (LAG-3, TIM-3, and TIGIT) and B7-family ligands alone or in association with first-series ICIs may be future promising approaches for RCC treatment. In addition, further preclinical or clinical studies need to assess the validity and applicability of prognostic biomarkers, which might help to personalize checkpoint combination therapy and ultimately increase the clinical response rate.

## Figures and Tables

**Table 1:** Novel immune checkpoint inhibitors: ongoing clinical trials in RCC.

Target	Drug	Clinical Trials
LAG-3	IMP231 Relatlimab	NCT00351949 NCT05148546
TIM-3	Sabatolimab INCAGN02390	NCT02608268 NCT03652077
VISTA	CA-170	NCT02812875
IDO1	Navoximod	NCT02471846

## Data Availability

No new data were created.
